# A rare case report of concurrent cryptococcal, streptococcal, and tuberculous meningitis in a patient with pulmonary tuberculosis

**DOI:** 10.1097/MD.0000000000040276

**Published:** 2024-10-25

**Authors:** Dewu Bi, Xiaolu Luo, Xike Tang, Xiaocheng Luo, Lida Mo

**Affiliations:** a Department of Clinical Laboratory, The Fourth People’s Hospital of Nanning, Nanning, China; b Key Laboratory of Infectious Diseases, The Fourth People’s Hospital of Nanning, Nanning, China; c Human Immunodeficiency Virus/Acquired Immunodeficiency Syndrome Clinical Treatment Center of Guangxi (Nanning), Nanning, China; d Department of Infectious Diseases, The Fourth People’s Hospital of Nanning, Nanning, China.

**Keywords:** case report, cryptococcal meningitis, pulmonary tuberculosis, *Streptococcal meningitis*, tuberculous meningitis

## Abstract

**Rationale:**

Meningitis caused by concurrent infections with *Cryptococcus neoformans*, *Streptococcus equi* subsp. *equi*, and *Mycobacterium tuberculosis* is extremely rare.

**Patient concerns:**

We present the case of a 63-year-old male patient who presented with headaches, dizziness, nausea, vomiting, and fever for the past 3 weeks.

**Diagnoses:**

The patient was diagnosed with concurrent cryptococcal, streptococcal, and tuberculous meningitis.

**Interventions:**

The patient received isoniazid, rifampicin, ethambutol, and levofloxacin for 1 month, in addition to liposomal amphotericin B with flucytosine for 2 weeks, followed by fluconazole with flucytosine for additional 2 weeks.

**Outcomes:**

The symptoms improved, and outpatient therapy was continued.

**Lessons:**

Infectious meningitis requires a combination of microscopy, culture, and rapid molecular diagnostics for early diagnosis and treatment.

## 1. Introduction

Meningitis can be caused by various microbes, including *Mycobacterium tuberculosis*, *Neisseria meningitidis* (*Meningococcus*), *Cryptococcus*, and *Streptococcus*.^[1–3]^ The use of immunosuppressants in acquired immunodeficiency syndrome,^[4]^ cancer,^[1]^ and organ transplantation,^[5]^ has increased the frequency of cryptococcal and tuberculous meningitis.

Tuberculous and cryptococcal meningitis are the most prevalent forms of chronic infectious meningitis,^[5–7]^ both of which can present with similar signs, symptoms, and cerebrospinal fluid (CSF) results. Given the vague clinical symptoms related to these conditions, tuberculous meningitis and cryptococcal meningitis are often misdiagnosed. Several studies have revealed the clinical characteristics that differentiate tuberculous meningitis from cryptococcal meningitis in human immunodeficiency virus (HIV)-infected individuals.^[1,8]^ However, early diagnosis and treatment of both cryptococcal and tuberculous meningitis, particularly tuberculous meningitis, remains a challenge for physicians. Delayed diagnosis and treatment are closely linked to poor outcomes.^[1,2]^

*Streptococcus equi* subsp. *equi*, an invasive bacterium with a narrow host range, belongs to the *Streptococcus zooepidemicus* group, a group C-hemolytic streptococcus that typically resides opportunistically in the upper airways of horses.^[3,9,10]^ While *S equi* subsp. *equi* rarely causes infections in humans, it has been linked to cases of bacteremia, septicemia, and meningitis in immunocompromised individuals. Most treatments involve beta-lactam antimicrobials^[9,11]^; however, streptococcal meningitis is associated with elevated mortality and morbidity rates.^[3,10]^

Cryptococcal and tuberculous meningitis are prevalent among individuals living with HIV^[1,2,12–14]^; however, these conditions can also occur in individuals with various forms of immunosuppressive and seemingly immunocompetent individuals.^[1,15]^ While these infections are prevalent among those living with HIV, they are exceedingly rare in persons without HIV. In this retrospective observational study, we present a case of effectively managed concurrent streptococcal, cryptococcal, and tuberculous meningitis in an individual without HIV but with tuberculosis.

## 2. Case presentation

**Figure 1.**
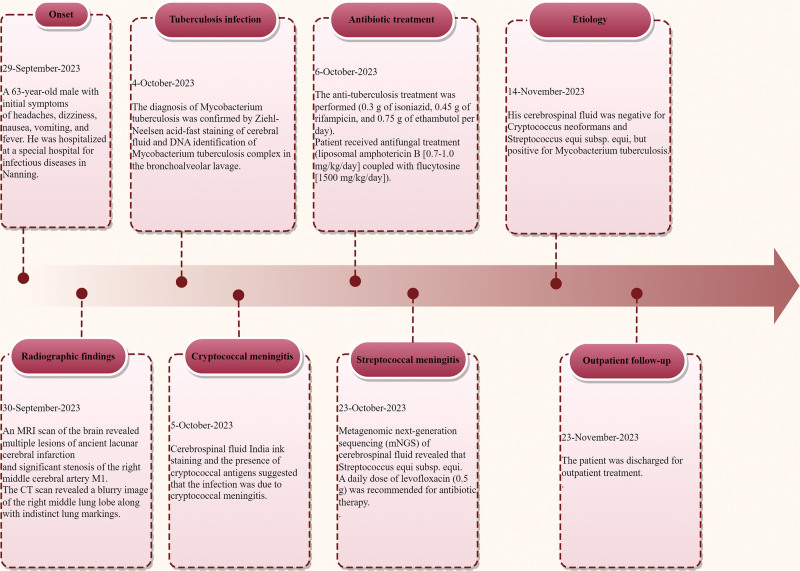
Timeline of the episode of care with relevant data.

A 63-year-old male with a history of cataract surgery in the right eye at the age of 58 and renal calculi for over 7 years received the Bacillus Calmette-Guerin vaccination schedule. He had no family history of hereditary diseases. The patient reported a 3-week history of headaches, dizziness, nausea, vomiting, and fever, with little explanation identified. He showed progressive weight loss over the past 6 months and tested negative for HIV. His wife and child were healthy, and neither he nor his family had a history of similar diseases. A timeline of the process, along with relevant data from the episode of care, is presented in Figure 1.

**Figure 2.**
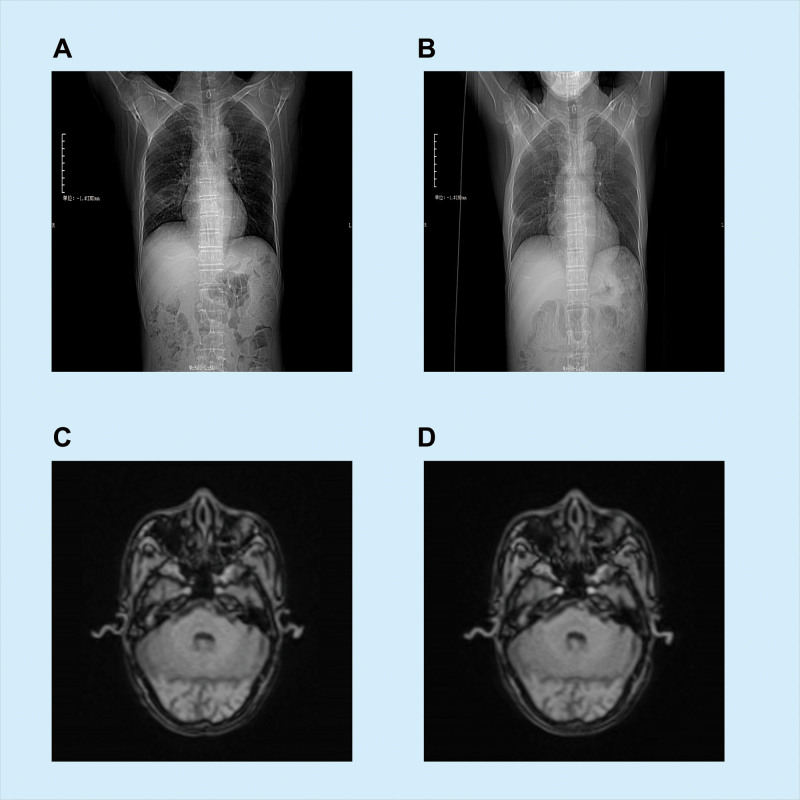
Radiological examination before and after treatment. (A) Chest CT before treatment. (B) Chest CT after treatment. (C) Pretreatment MRI examinations of the patient. (D) Posttreatment MRI examinations of the patient. CT = computed tomography, MRI = magnetic resonance imaging.

Brain magnetic resonance imaging (MRI) and magnetic resonance angiography revealed multiple lesions of ancient lacunar cerebral infarction in the bilateral corona radiata and the fronto-temporo-parietal area (Fig. 2A and B). The MRI/magnetic resonance angiography also revealed significant stenosis in the M1 segment of the right middle cerebral artery and the internal carotid artery owing to arteriosclerosis and right mastoiditis. A contrast-enhanced chest computed tomography scan indicated indistinct lung markings and prominent ground-glass opacities with a blur in the right middle lung lobe (Fig. 2C and D). Ultrasonography confirmed the presence of kidney stones.

**Figure 3.**
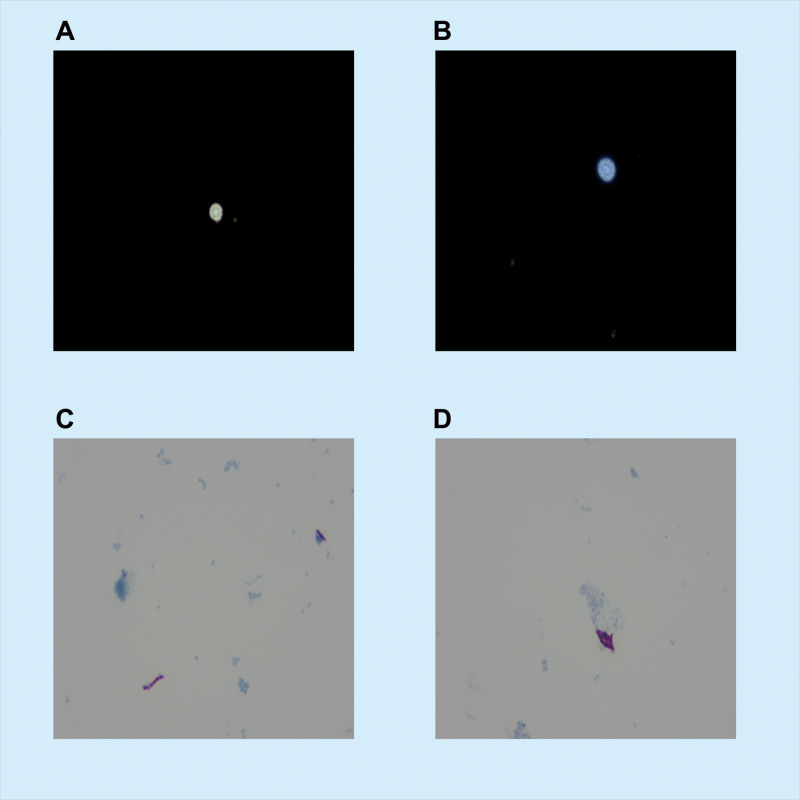
Aetiological examinations of cerebrospinal fluid (India Ink stain and Ziehl-Neelsen acid-fast stain). (A, B) Aetiological investigation using high magnification (×400) light microscopy. Following the India ink staining, the *Cryptococcus* can be identified in cerebrospinal fluid. (C, D) The Ziehl-Neelsen acid-fast staining of the patient’s cerebrospinal fluid in the etiological investigation revealed a positive acid-fast stain (×1000 magnification).

Table 1 summarizes the findings of CSF examination before and after treatment. Subsequent CSF analysis indicated increased protein levels with leukocytosis; however, cryptococcal antigens and India ink stains were also positive (Fig. 3A and B). *Cryptococcus neoformans* was identified using matrix-assisted laser desorption ionization-time-of-flight mass spectrometry (MALDI-TOF MS) following a positive culture for *Cryptococcus* from the CSF. Metagenomic next-generation sequencing of the CSF revealed the presence of *S equi* subsp. *equi*. Over a month prior, the patient had traveled to grasslands and made contact with horses. *M tuberculosis* was verified by Ziehl-Neelsen acid-fast staining of the CSF and deoxyribonucleic acid identification of the *M tuberculosis* complex in bronchoalveolar lavage fluid (Fig. 3C and D). *M tuberculosis* is typically confirmed by culturing bronchoalveolar lavage fluid, while polymerase chain reaction positivity for *M tuberculosis* confirms the diagnosis of tuberculous meningitis.

**Table 1:** The results of cerebrospinal fluid and blood examination before and after treatment.

	Pretreatment	Posttreatment	Reference intervals
Cerebrospinal fluid			
Leukocytes (×10^9^/L)	0.064	0.057	0.00–0.01
Mononuclear cells (%)	70.3	94.8	—
Glucose (mmol/L)	0.02	0.04	2.5–4.5
Chloride (mmol/L)	116.5	115	120–132
Total protein (mg/L)	1725	1474	150–450
Lactate dehydrogenase (U/L)	48	36	0–40
Adenosine deaminase (U/L)	1.8	0.9	0–8
Venous blood samples			
CD4+ T cells (/µL)	491	560	387–1350
CD8+ T cells (/µL)	248	299	247–1077
CD45+ T cells (/µL)	914	1088	1187–3835
White blood cell (×10^9^/L)	11.2	3.1	3.5–9.5
Red blood cell (×10^12^/L)	3.9	2.5	4.3–5.8
Hemoglobin (g/L)	114	76	130–175
Platelet (×10^9^/L)	231	158	125–350
Neutrophils (×10^9^/L)	9.8	1.3	1.8–6.3
Lymphocytes (×10^9^/L)	0.8	1.4	1.1–3.2

The patient underwent a triple therapy regimen for tuberculosis and *S equi* subsp. *equi,* which included 0.3 g of isoniazid, 0.45 g of rifampicin, and 0.75 g of ethambutol daily, along with antibiotics, and levofloxacin (0.5 g daily) for 1 month. The antifungal induction therapy included 2 weeks of liposomal amphotericin B (0.7–1.0 mg/kg/d) combined with flucytosine (1500 mg/kg/d). This was later switched to fluconazole (300 mg/kg/d) combined with flucytosine (1500 mg/kg/d) due to impaired kidney function developed during treatment with liposomal amphotericin B. Polymerase chain reaction assays and microbiological etiology revealed no evidence of bacterial or fungal infection. After 4 weeks, all clinical symptoms and signs improved significantly.

## 3. Discussion

The present case report highlights a rare diagnosis of concurrent cryptococcal, streptococcal, and tuberculous meningitis in a patient without HIV but with pulmonary tuberculosis, which has not yet been documented. Consistent with previous studies,^[1,15]^ cryptococcal meningitis and tuberculous meningitis occur simultaneously in individuals who appear to be immunologically competent. However, this case depicts a typical meningitis caused by 3 separate organisms: *C* neoformans, *S equi* subsp. *equi*, and *M tuberculosis*. We present a case of an effective clinical management intervention. Furthermore, the patient in this study was fully immunocompetent, with a normal hemocyte count, and tested negative for HIV infection.

In this study, the patient presented with headache as the initial symptom, which is consistent with previous studies that identify headache as the most frequent clinical manifestation of cryptococcal and tuberculous meningitis.^[1,16–21]^ In both types of meningitis, headache is a prognostic factor, with its absence associated with a poor prognosis. This correlation may be due to the inflammatory processes occurring in the subarachnoid space during meningitis, which contribute to the development of headaches.^[1,21]^

*S equi* subsp. *equi*, a group C *Streptococcus* primarily linked to zoological infections, is a rare human pathogen.^[3,9,10]^ According to previous studies,^[3,9–11,22–24]^ most cases of infection have been attributed to *S equi subsp*. *zooepidemicus*, with only 2 confirmed instances caused by *S equi* subsp. *equi*, both of which occurred in children. Thus, the patient of interest is the first adult diagnosed with meningitis caused by *S equi* subsp. *equi*.

While infections caused by *S equi* subsp. *zooepidemicus* and *S equi* subsp. *equi* have been reported, cryptococcal and tuberculous meningitis have not been documented in individuals with streptococcal meningitis. To the best of our knowledge, this is the first case of a patient with pulmonary tuberculosis who successfully received diagnosis and treatment for concomitant cryptococcal, streptococcal, and tuberculous meningitis. The 2 most prevalent infections among individuals living with HIV are tuberculous and cryptococcal meningitis, with cryptococcal meningitis being the leading cause of mortality in developing nations.^[1,4,25]^ Additionally, individuals with different types of immunodeficiency and those who appear to be immunocompetent, can develop cryptococcal and tuberculous meningitis.^[1,15]^ Proper diagnosis and early treatment may contribute to reduced mortality rates linked to cryptococcal and tuberculous meningitis.

While the physical signs and symptoms of meningitis are similar, the diagnosis is complex and mostly relies on radiological investigations. During the initial evaluation of the CSF, the patient was found to have cryptococcal, streptococcal, and tuberculous meningitis. The management of streptococcal meningitis is based on beta-lactam antibiotics.^[9,11]^ Given the *C neoformans*, *S equi* subsp. *equi* and *M* tuberculosis infections, the patient was treated for 4 weeks with isoniazid, rifampicin, ethambutol, and levofloxacin, along with concomitant liposomal amphotericin B and flucytosine. The enhanced synergistic benefits of this treatment regimen were demonstrated in vivo, with fewer adverse effects.

## 4. Conclusion

To our knowledge, this is the first report of concurrent cryptococcal, streptococcal, and tuberculous meningitis in a patient diagnosed with pulmonary tuberculosis. Early diagnosis and effective antibiotic therapy are crucial, as both can significantly improve patient outcomes. This rare occurrence of meningitis provides valuable insights into the use of numerous clinical assays to achieve precision medicine and personalized therapy.

## Acknowledgments

Thank you to all colleagues who participated in medical treatment.

## Author contributions

**Conceptualization:** Dewu Bi, Lida Mo.

**Writing – original draft:** Dewu Bi.

**Supervision:** Xiaolu Luo, Xike Tang.

**Validation:** Xike Tang.

**Data curation:** Xiaocheng Luo.

**Investigation:** Xiaocheng Luo.

**Project administration:** Lida Mo.

**Resources:** Lida Mo.
